# Integration as a legacy of Africa’s mpox response

**DOI:** 10.1371/journal.pgph.0004932

**Published:** 2025-07-16

**Authors:** Ngashi Ngongo, Abou Beck Gaye, Gervais Folefack, Nebiyu Dereje, Jean Kaseya, Yap Boum

**Affiliations:** 1 Africa Centres for Disease Control and Prevention (Africa CDC), Addis Ababa, Ethiopia; 2 World Health Organization, Brazzaville, Congo; PLOS: Public Library of Science, UNITED STATES OF AMERICA

The mpox outbreak in Africa has highlighted the limitations of public health systems and revealed critical gaps in epidemic response. However, it has also provided a unique opportunity to redesign and integrate response mechanisms, with potential benefits extending beyond mpox to enhance Africa’s overall pandemic preparedness and response. Following the declaration of mpox as a public health emergency of continental security and international concern in August 2024, the response to the emergency was designed as one team, one plan, one budget, and one M&E framework under the umbrella of the continental Incident Management System Team (IMST). This approach has brought all the relevant partners together, avoided duplication, and increased synergy [1]. The IMST developed the first Mpox Continental Response Plan, which had 10 strategic response pillars, including leadership and coordination, surveillance, vaccination, laboratory, research, risk communication and community engagement (RCCE), continuity of essential healthcare services, case management, infection prevention and control (IPC), and logistics and supply chain [2]. This continental plan was implemented from September 2024 to February 2025.

At the end of the six-month implementation period, the Emergency Consultative Group (ECG) reconvened to evaluate the epidemiologic situation and response progress of mpox and recommended intensification of the response efforts while ensuring strengthening of the overall health system. This recommendation led to the development of Mpox Continental Response Plan 2.0 [3], led by Africa CDC and WHO, with more than 28 partners, emphasizing integration as a central legacy of this crisis, with a clear vision to strengthen Africa’s outbreak preparedness and response architecture.

At the heart of this vision is the Incident Management System Team (IMST), which is transitioning from a vertical, disease-specific response mechanism into a cross-cutting coordination model capable of simultaneously managing multiple outbreaks, including cholera, measles, anthrax, Marburg and mpox. By integrating disease-specific expertise, resources and operations under a unified command, the IMST model has the potential for greater equity, efficiency, and accountability in the current global health architecture with reduced resources in the current global health reduced funding [4]. This collaborative structure, co-led by Africa CDC and WHO AFRO and supported by humanitarian and development partners, may well become the future blueprint for the outbreak response in Africa.

Beyond coordination, syndromic integration has emerged as a pragmatic strategy to address multiple concurrent outbreaks with similar symptoms, especially when laboratory resources are limited. The African health system is characterized by a limited skilled health workforce and infrastructure. To bridge this gap in diagnostics capacity, the mpox response has catalyzed the adoption of multiplex testing platforms that allow simultaneous screening for mpox, measles, and varicella (chickenpox), conditions that often co-circulate and mimic one another clinically [5]. This approach is vital in fragile areas such as Eastern DRC, where misdiagnosis can lead to delayed containment and misallocation of resources, underscoring the need for the development of integrated diagnostic approaches to address the conditions. Moreover, as these conditions share similar clinical presentations, a syndromic integration improves diagnostic accuracy, case management and survival, ensuring that health systems respond holistically, rather than through fragmented, disease-specific silos [6,7].

Crucially, integration has extended to national response frameworks, with many countries now embedding mpox surveillance and case management within their broader disease control programs. Vaccination strategies are adapting to include pediatric populations aged 1–17 years based on emerging epidemiological data. National immunization programs are incorporating mpox vaccination of populations one year and above in accordance with Immunization Agenda 2030 [8]. In parallel, mpox has been positioned as a compelling case for local manufacturing of mpox diagnostics and vaccines, reinforcing the urgency of African-led health product production. This strategic alignment with the African Union’s Platform for Harmonized African Health Products Manufacturing (PHAHM) marks a critical shift from emergency response to long-term health sovereignty [9].

Despite ongoing challenges, particularly in terms of financing and equitable vaccine access, the mpox response has catalyzed the transformation of emergency preparedness and response in Africa. This transformation has expedited integration across various domains, including governance, coordination, diagnostics, clinical management, and manufacturing. The impact of mpox in Africa may ultimately be measured not just by its immediate effects on the virus but by the systemic enhancements it has fostered. If these integrated approaches are maintained and supported, they could signify a pivotal shift towards a new model for public health in Africa, where each outbreak presents an opportunity for broader systematic strengthening [10].
